# Synthesis and Conformational Characteristics of Thermosensitive Star-Shaped Six-Arm Polypeptoids

**DOI:** 10.3390/polym12040800

**Published:** 2020-04-03

**Authors:** Tatyana Kirila, Anna Smirnova, Alla Razina, Andrey Tenkovtsev, Alexander Filippov

**Affiliations:** Institute of Macromolecular Compounds of the Russian Academy of Sciences, Bolshoy pr., 31, 199004 Saint Petersburg, Russia; av.smirnova536@gmail.com (A.S.); allarazina@yahoo.com (A.R.); avt@hq.macro.ru (A.T.); afil@imc.macro.ru (A.F.)

**Keywords:** star-shaped polypeptoids, poly-2-alkyl-2-oxazines and poly-2-alkyl-2-oxazolines, synthesize, molecular hydrodynamics and optics, conformational and hydrodynamic characteristics, thermosensitivity

## Abstract

Star-shaped six-arm poly-2-alkyl-2-oxazine and poly-2-alkyl-2-oxazoline with hexaaza [2_6_]orthoparacyclophane derivative core were synthesized successfully using cationic ring-opening polymerization. Conformational behavior of prepared polymer stars were investigated by the methods of molecular hydrodynamics and optics in molecular dispersed solutions. It was shown that conformation characteristics of star-shaped polypeptoids depends on arm length, while the chemical structure weakly affects the behavior of the studied polymers in solutions. This behavior is caused by the close equilibrium rigidity of arms. The star-shaped polypeptoids have relatively high intramolecular density. All synthesized stars exhibit LCST behavior. Phase separation temperature depends on arm structure. It is lower for poly-2-alkyl-2-oxazines, monomer units of which contains one methylene group more than monomers of poly-2-alkyl-2-oxazoline.

## 1. Introduction

At present star-shaped polymers of amphiphilic nature are the subject of intensive research, since such systems are considered as promising structures for drug delivery systems and other biomedical applications [1,2]. It was shown that transition from linear to star-shaped macromolecules significantly increases the polymer’s ability of molecular recognition [3,4]. Special attention is paid to the derivatives of polyethylene oxide and polyalkyloxazolines, which is resulting from their biocompatibility and the ability to show low critical solution temperature (LCST) behavior, which allows controlling the processes of complexation of low-molecular compounds [5,6,7,8]. It should be pointed out that incorporation into the polymer of such moieties as macrocycles, for example calixarenes, cyclodextrins, etc., leads to a significant increase of the affinity of these macromolecules to low-molecular compounds and metal ions.

Star-shaped polymers with an azacyclophane core have not been described so far due to the low synthetic availability of parent macrocycles. On the other hand, hexaaza[2_6_]orthoparacyclophanes can be obtained through preparative yields by reduction of the corresponding macrocyclic Schiff bases, which can be easily prepared by cyclocondensation of aromatic dialdehydes with diamines [9,10]. It was found [11,12,13,14] that these compounds and their hydrogenated derivatives (macrocyclic polyamines) form stable complexes of a certain stoichiometry with various aromatic compounds and metal ions. These complexes exhibit higher stability in comparison with the similar ones based on calixarene type macrocycles [11]. Functionalization of macrocycle amino groups by methods developed by the authors during the elaboration the synthetic approaches to star-shaped polymers based on calix[8]arene [15], makes it possible to obtain polyfunctional initiators suitable for producing star-shaped poly-2-alkyl-2-oxazines (PAlOz) and poly-2-alkyl-2-oxazolines (PAlOx)—the new classes of water-soluble polymers with high affinity to aromatic compounds of various natures and complexes based on them.

To date, a large number of PAlOx have been obtained, including statistical, block and gradient copolymers, grafted and star-shaped polymers [16,17,18,19,20]. The polymerization processes are studied in detail, in particular, the kinetics of oxazolines polymerization that have been initiated by alkyl halides, tosylates, nosylates and triflates [21,22]. For PAlOx, the relations between their chemical structure and the behavior in aqueous solutions were established and features distinguishing them from other thermosensitive polymers were revealed [23,24,25,26]. It is shown that the insertion into the polymer of special moieties that are capable of effective binding with inorganic ions and organic compounds is a promising strategy for the construction of supramolecular colloidal structures, which allow simulating the processes occurring in nature [27]. For star-shaped PAlOx, the influence of the length and number of arms on the processes of self-organization and aggregation was established [28,29,30].

Homologues of PAlOx, namely, poly-2-alkyl-2-oxazines have not been studied, although it is well known that they can be obtained by cationic ring-opening polymerization [31]. However, the process is characterized by low polymerization rate constants and high rate of chain transfer, which makes it difficult to obtain high-molar-mass samples [32,33,34]. This is one of the reasons for the small number of papers devoted to the study of this promising class of thermosensitive polymers. Poly-2-ethyl-2-oxazine (PEtOz) and poly-2-isopropyl-2-oxazine (PiPrOz) have been shown to be water-soluble thermosensitive polymers [35,36]. As for PAlOx, hydration of PAlOz determines the structure of the lateral moieties [37,38]. In particular, by changing the substitute structure in the 2-oxazine monomer, it is possible to obtain a hydrophilic or lipophilic polymer or a combination of two types of monomers—amphiphilic block copolymers [39]. The additional methylene group in the main chain makes PAlOz more hydrophobic in comparison with PAlOx having the same lateral alkyl radical, which leads to the decrease in turbidity temperatures of their aqueous solutions [33]. The most significant is the fact that PAlOz have much higher binding ability to incorporate water—insoluble drugs in comparison with PAlOx [38]. This indicates a very good prospects for using PAlOz in medicine, but, unfortunately, studies of this class of polymers are just at the initial stage. In the literature, data on the synthesis of PAlOz of complex architecture are few [40].

In this study, new six-arm polymer stars with PAlOx and PAlOz arms were prepared by cationic ring-opening polymerization (CROP). The derivative of hexaaza[2_6_]orthoparacyclophane (CPh6) was a multicenter initiator. Accordingly, we examined the star-shaped poly-2-ethyl-2-oxazine (CPh6-PEtOz), poly-2-isopropyl-2-oxazine (CPh6-PiPrOz), poly-2-ethyl-2-oxazolines (CPh6-PEtOx), and poly-2-isopropyl-2-oxazolines (CPh6-PiPrOx). For molar mass characterization and conformational studies, we employed static and dynamic light scattering in combination with viscometry in molecularly dispersed solutions. The LCST behavior was tested by light scattering and turbidimetry.

## 2. Materials and Methods

### 2.1. Polymer Star Synthesis

The solvents and reagents (all Sigma-Aldrich, St. Louis, MO, USA) were purified and dried according to the standard techniques. Trianglamine (1) [11] as well as 2-alkyl-2-oxazolines and 2-alkyl-2-oxazines [41] were synthesizes by the generally applied methods.

#### 2.1.1. Preparing of Hexa-N-(11-bromoundecanoyl) Trianglamine (2)

A flask equipped with a stirrer and a gas-supplying tube was charged with **1** (0.13 g, 2 × 10^−4^ mol), Cs_2_CO_3_ (0.5 g, 2.4 × 10^−3^ mol), and acetonitrile (8 mL). The mixture was cooled up to 0 °C, and the solution of 11-bromoalkanoic acid chloride (0.69 g, 2.4 × 10^−3^ mol) in acetonitrile (3 mL) was added under intense stirring. The mixture was allowed to stay at room temperature for two days diluted with water (20 mL), extracted by methylene chloride and dried over magnesium sulfate. The product was purified by flash chromatography silica 100/160 mesh, ethyl acetate/hexane 1:10). Yield 0.45 g (56%). ^1^H NMR (CDCl_3_): 7.10 (s), 5.05 - 4.9 (m), 4.07 (t), 3.43 (t), 2.47 (t), 2.34 (m), 1.87 (quint), 1.67 (m), 1.31–1.27 (m).

NMR spectrum of the initial macrocycle is given in Supplementary Materials (Figure S1).

#### 2.1.2. Polymerization of Oxazolines(Oxazines) Using 2 As Initiator. Typical Procedure

An ampoule containing the desired amount of initiator (of about 200 mg), the appropriate amount of 2-alkyl-2-oxazine or 2-alkyl-2-oxazoline (initiator: monomer = 1: 30 in terms of functional groups), and 3 mL of sulfolane was frozen to −196 °C, air was removed under vacuum (0.1 mm Hg), and the mixture was thawed in argon atmosphere. The cycle was repeated three times, then the ampoule was sealed and heated at 100 °C for 72 h. Further 50% aqueous ethanol (1 mL) was added, and the resulting mixture was allowed to stay at room temperature for 24 h. The reaction mixture was dialyzed against water for 24 h, and lyophilized.

Star-shaped poly-2-isopropyl-2-oxazoline: ^1^H NMR (CDCl_3_): δ ppm. 4.16 (m, core) 3.88 (m, core), 3.37 (m, core), 3.47 (br.s CH_2_CH_2_N), 2.92, 2.68 (br. s CH(CH_3_)_2_, rotamers), 1.61 (m, core), 1.29 (m, CH_3_ + core).

Star-shaped poly-2-isopropyl-2-oxazine: ^1^H NMR (CDCl_3_): δ ppm. 4.20 (m, core) 3.88 (m, core), 3.33 (m CH_2_CH_2_CH_2_N), 2.72 (br. s CH(CH_3_)_2_, 1.80 (m CH_2_CH_2_CH_2_N) 1.85 (m, core), 1.52 (m, core) 1.29 (m, CH_3_ + core).

Star-shaped poly-2-ethyl-2-oxazoline: ^1^H NMR (CDCl_3_): δ ppm. 4.56 (m, core) 3.96 (m, core), 3.73 ((m, core), 3.45 (br.s CH_2_CH_2_N), 2.42, 2.32 (br. s CH(CH_3_)_2_, rotamers), 1.61 (m, core), 1.29 (m, CH(CH_3_)_2_ + core).

Star-shaped poly-2-isopropyl-2-oxazine: ^1^H NMR (CDCl_3_): δ ppm. 4.11 (m, core) 3.31 (m CH_2_CH_2_CH_2_N+core), 2.31 (br. s CH(CH_3_)_2_, 2.07 (m, core), 1.80 (m CH_2_CH_2_CH_2_N) 1.52 (m, core) 1.29 (m, CH_3_ + core).

NMR spectra of the prepared polymer stars are given in Figure S2.

### 2.2. Solution Investigation

The molar masses (MM) of the synthesized stars were obtained by sedimentation-diffusion analysis in dilute solutions in chloroform (density ρ = 1.486 g⋅cm^−3^, dynamic viscosity η_0_ = 0.57 cP, and refractive index n_0_ = 1.446). The velocity sedimentation experiments were performed on the MOM-3180 analytical ultracentrifuge (Budapest, Hungary). The rotor rotation speed was 45,000 rpm. The sedimentation pattern was recorded by the Philpot–Svensson refractometric optical system. The experimental data were processed using the GetData Graph Digitizer program (vers. 2.24). Sedimentation coefficients *s* were calculated from the velocity of the sedimentation boundary in the concentration range *c* = 0.0019–0.0147 g⋅cm^−3^. The concentration dependences of *s* are satisfactorily described by the Gralen relationship

**Figure 1:**
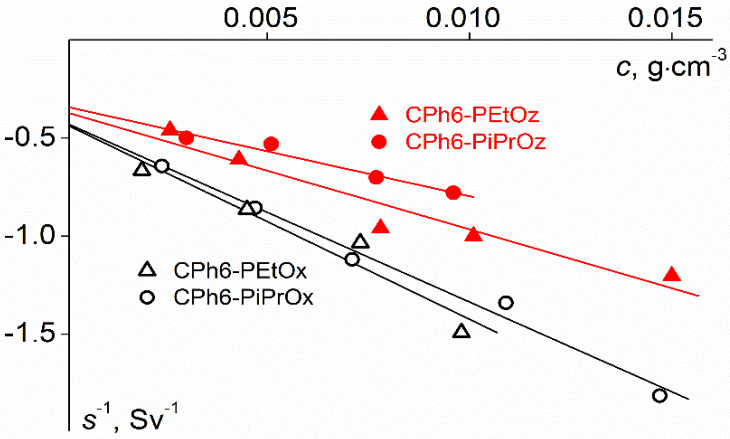
Concentration dependences of the inverse sedimentation coefficient 1/*s* for the studied polymer stars in chloroform.

1/*s* = 1/*s*_0_(1 + *k*_s_*c*), (1)

where *s*_0_ is the sedimentation constant and *k*_s_ is the sedimentation coefficient (Figure 1). The *k*_s_ values of the investigated stars were within 11–27 cm^3^ g^−1^. Table 1 lists the *s*_0_ values.

**Figure 2:**
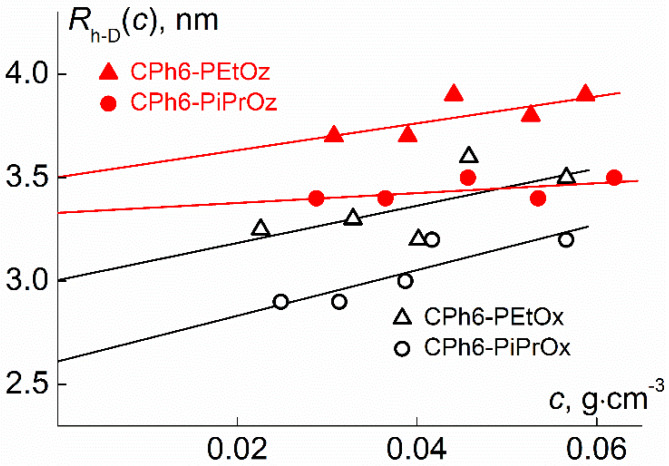
Plots of *R*_h-D_(*c*) vs. concentration *c* for the studied polymer stars in chloroform.

The hydrodynamic radii *R*_h-D_ of macromolecules and the translational diffusion coefficients *D*_0_ = *k*_Б_*T*/6πη_0_*R*_h-D_ were obtained by dynamic light scattering using the Photocor Complex (Photocor Instruments Inc., Moscow, Russia). The light source was the Photocor DL diode laser with a wavelength λ = 658.7 nm. The correlation function of the scattered light intensity was obtained using the Photocor-PC2 correlator with the channel number of 288 and processed using DynalS software (ver. 8.2.3, SoftScientific, Tirat Carmel, Israel). Figure 2 shows the dependences of hydrodynamic radii *R*_h-D_(*c*) obtained at concentration *c*. The change in *R*_h-D_(*c*) reflects the concentration dependence of the diffusion constant D. Extrapolation of *R*_h-D_(*c*) to *c* = 0 yields the hydrodynamic radius *R*_h-D_ of the macromolecules (Table 1).

**Figure 3:**
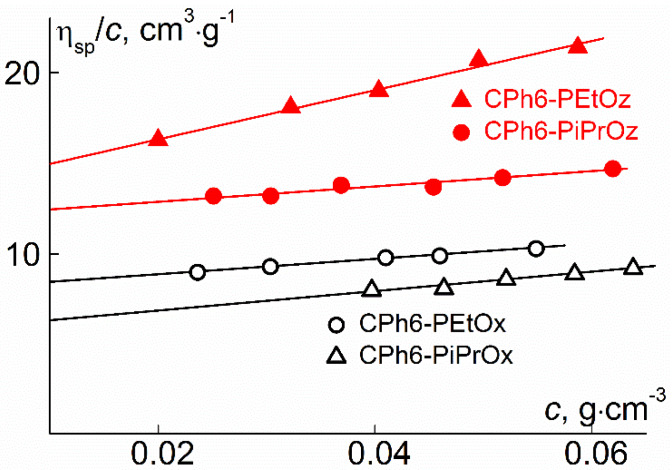
Reduced viscosity η_sp_/*c* vs. *c* for the studied polymer stars in chloroform.

The so-called hydrodynamic molecular masses *M*_sD_ for CPh6-PAlOx and CPh6-PAlOz were calculated using the Svedberg equation:

(2) $$M_{\mathrm{sD}}=\frac{RT}{1-\bar{v}\rho }\frac{s_{0}}{D_{0}}$$

where *R* is the universal gas constant and *T* is the absolute temperature. The specific partial volume $\bar{v}$ was determined using a densitometer (Density/Specific Gravity Meter DA-640, KEM, Tokyo, Japan). The values of $\bar{v}$ for investigated stars differed slightly (Table 1). The obtained values of $\bar{v}$ for the CPh6-PEtOx are close to those reported in the literature for linear PEtOx, to reported values from Schubert and Nischang et al. [26] of $\bar{v}$ = 0.84 cm^3^g^−1^, from Ye et al. [42] of $\bar{v}$ = 0.85 cm^3^ g^−1^, and $\bar{v}$ = 0.87 cm^3^g^−1^ from Chen et al. [43]. In addition, the specific partial volumes for CPh6-PAlOx are in good agreement with the values of $\bar{v}$ for star-shaped PEtOx and PiPrOx [44,45,46]. MMs calculated by the relation (2) are shown in Table 1, which also gives the values of intrinsic viscosity [η]. The intrinsic viscosity was measured with the Ostwald-type Cannon–Manning capillary viscometer (Cannon Instrument Company Inc., State College, PA, USA) at 21 °C. The solvent efflux time was 48.5 s. Figure 3 shows reduced viscosity η_sp_/*c* versus concentration for solutions of synthesized stars. These dependences were analyzed using the Huggins equation

η_sp_/*c* = [η] + *k*′ [η]^2^*c* (3)

where *k*′ is the Huggins constant characterizing the polymer-solvent hydrodynamic interaction and the hydrodynamic behavior of solutions [47,48,49]. In most cases, the Huggins constants turned out to be high (*k*′ > 0.5). The increased values of *k*′ were found for polymer brushes, hyperbranched and star-shaped polymers [50,51,52,53,54]. It is possible that this phenomenon is typical for solutions of compact symmetric particles. For star-shaped CPh6-PAlOx and CPh6-PAlOz parameter γ = *k*_s_/[η] is appreciably above γ =1.7, a value that is typical for linear flexible-chain polymers in good solvents [47,55]. Interestingly, the γ value increases with the increase in intramolecular density. In fact, for rigid-chain polymers, parameter γ is markedly lower than 1.7, sometimes by almost an order of magnitude [47].

The refractive index increment *dn*/*dc* was determined using the RA-620 refractometer (KEM, Japan). The index *dn*/*dc* for CPh6-PAlOx is similar to that for star-shaped PEtOx and PiPrOx with another structure of core [20,46]. Note that the refractive index increments for CPh6-PAlOx are higher than *dn*/*dc* for their axozine analogs. Besides, the *dn*/*dc* values decrease on passage from polymers with ethyl side groups to stars containing isopropyl.

The LCST behavior of the synthesized samples in water was studied by turbidimetry using the Photocor Complex. Measurements were carried out at the concentration *c* = 0.0015 g⋅cm^−3^. The experiments were performed in the temperature range from 15 to 75 °C. The temperature was changed discretely with a step from 0.5 to 5 °C and regulated with the precision of 0.1 °C.

## 3. Results and Discussion

### 3.1. Polymer Synthesis And Structural Characteristics

**Figure 4:**
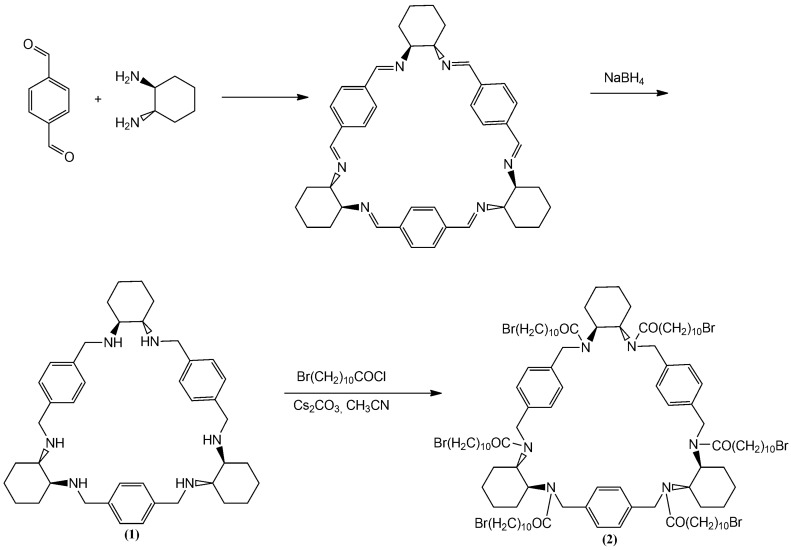
Scheme of synthesize of multicenter initiator.

The parent ((2R, 3R, 12R, 13R, 22R, 23R)-1,4,11,14,21,24-Hexaaza-(2,3:12,13:22,23)-tributeno-(6,9:16,19:26,29) - trietheno - 2H,3H,12H,22H,23H- (30))—annulene (1), known under the trivial name of “trianglamine”, was obtained by the well-known method [56] cyclocondensation of terephthalic aldehyde with (1R, 2R)-trans-diaminocyclohexane followed by the Schiff base reduction with sodium borohydride (Figure 4). Acylation of the resulting hexamine with 11-bromundecanoyl chloride in the presence of caesium carbonate leads to the corresponding hexamide formation, which was used as a macrocyclic initiator of the cationic polymerization of 2-alkyl-2-oxazolines and 2-alkyl-2-oxazines.

**Figure 5:**
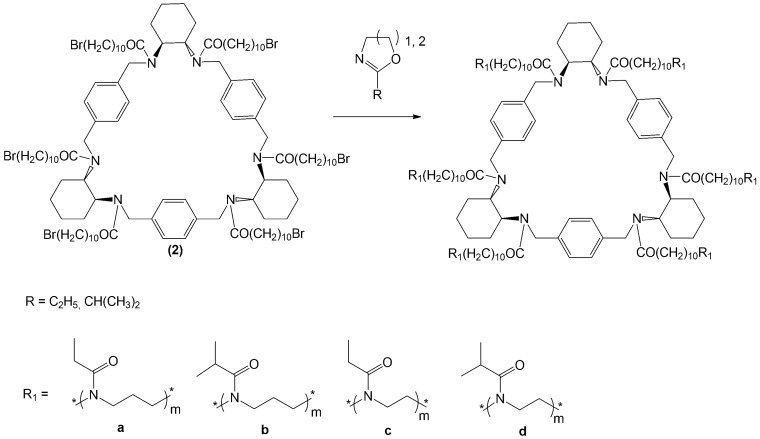
Scheme of synthesize of star-shaped six-arm CPh6-PEtOz (**a**), CPh6-PiPrOz (**b**), CPh6-PEtOx (**c**), and CPh6-PiPrOx (**d**).

It should be noted that other amidation procedures, in particular using different tertiary amines as bases (pyridine, triethylamine, 4-dimethylaminopyridine) led to incomplete acylation of cyclophane, which is probably the result of non-covalent interactions of the acceptor and the macrocycle. CROP was performed using the method developed by the authors for the synthesis of star-shaped poly-2-alkyl-2-oxazolines [57] and poly-2-alkyl-2-oxazines [36,40] with the calixarene core. It was found that the most suitable solvent for polymerization using abovementioned initiators is sulfolan. The synthesis scheme for CPh6-PAlOx and CPh6-PAlOz is shown in Figure 5.

**Figure 6:**
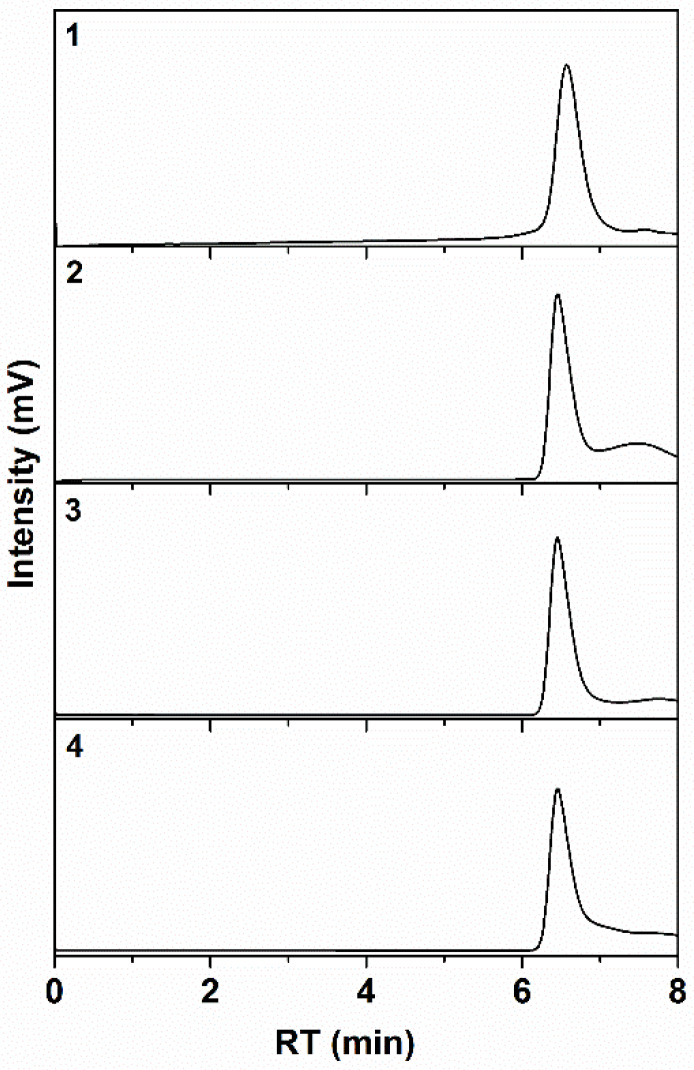
GPC curves of star-shaped CPh6-PEtOx (**1**), CPh6-PiPrOx (**2**), CPh6-PEtOz (**3**), and CPh6-PiPrOz (**4**).

Unfortunately, there are no effective procedures that give the possibility to achieve the selective cleavage of amide bond between the core and arms without destruction the very similar amide moieties in polyoxazoline fragments in the polymers under investigation. In order to verify the number of the arms we used the well-known approach based on correlation between molecular mass and hydrodynamic radii of star shaped polymers [58]. In Figure 6, the chromatograms of the star-shaped CPh6-PAlOx and CPh6-PAlOz are given. Monomodality and symmetrical form of the curve indicates the uniform structure of the polymers. In addition, all SEC traces have symmetrical forms that indicate the absence in the samples both higher- molecular and lower- molecular fractions. This suggests that the overwhelming number of CPh6 -PAlOx and CPh6-PAlOz under investigation have six arms while the number of “defective” five-arms as well as four-arms molecules is small.

The difference in the MMs of oxazoline and oxazine stars results in the difference in the content of hydrophobic groups ω in their macromolecules (Table 2). In the studied polymers, the hydrophobic components include the CPh6 core and –(CH_2_)_10_– chains. For CPh6-PAlOx, the ω value is about 11 mol %, that is almost three times lower than ω for star-shaped PiPrOx with the carbosilane dendrimer as the core [46]. For PAlOx with calix[n]arene core, depending on the arm length, the molar fraction of hydrophobic groups is either close to or higher than the ω value for CPh6-PiPrOx [30,44]. CPh6-PAlOz are characterized by very low ω values, which are noticeably lower than ω = 28 mol% for four-beam PEtOz with calix[4]arene core [40].

The discussed differences in ω values are due to the structure of the branching center and the arm length. CPh6-PAlOx and CPh6-PAlOz have a relatively low core MM (*M*_core_ = 810 g⋅mol^−1^), while *M*_core_ = 1310 and 9048 g⋅mol^−1^ for calix[8]arene and the second-generation carbosilane dendrimer, respectively. As for the *L*_arm_ arm length, it is close or greater for the studied polymers than the *L*_arm_ of the star-shaped PAlOx and PAlOz with calix[n]arene or dendrimer core [30,40,44,46]. The polymerization degree *N*_tsc_ of the thermosensitive chains for CPh6-PAlOx and CPh6-PAlOz was calculated by the ratio:

*N*_tsc_ = (*M*_sD_ − *M*_core_ − 6⋅*M*_alk_)/6*M*_0,_ (4)

where *M*_0_ is the MM of PAlOx and PAlOz monomer units and *M*_alk_ = 140 g⋅mol^−1^ is the MM of alkylene chains. The lengths *L*_tsc_ of the thermosensitive chains and *L*_arm_ of the arms were calculated under the assumption that all valence bonds have the same length of 0.14 nm, and the valence angles are tetrahedral. Then the length of the monomer unit is λ_0_ = 0.378 nm for PAlOx and 0.504 nm for PAlOz, and the length of the –(CH_2_)_10_– chain is 1.26 nm. Table 2 shows that the PiPrOx and PEtOx chains are almost two times smaller than *L*_tsc_ for PiPrOz and PEtOz, respectively. Notably, in both pairs of stars the length of the PiPrOx and PiPrOz chains is less than the length of the PEtOx and PEtOz chains.

### 3.2. Hydrodynamic Characteristics And Conformation of CPh6-PAlOx And CPh6-PAlOz Macromolecules

For all the polymers studied, the arm length *L*_arm_ is much larger than the hydrodynamic radius of their macromolecules (Table 2 and Table 3). The *L*_arm_/*R*_h-D_ ratio ranges from 3.2 to 4.9. These facts are sufficient to indicate that the macromolecules are compact, and the arms are quite strongly folded. More rigorous conclusions about the conformation of CPh6-PAlOx and CPh6-PAlOz macromolecules can be made by comparing their hydrodynamic characteristics with the data for linear PAlOx. (Unfortunately, we are not aware of any works devoted to the study of the hydrodynamic and conformational properties of PAlOz.)

**Figure 7:**
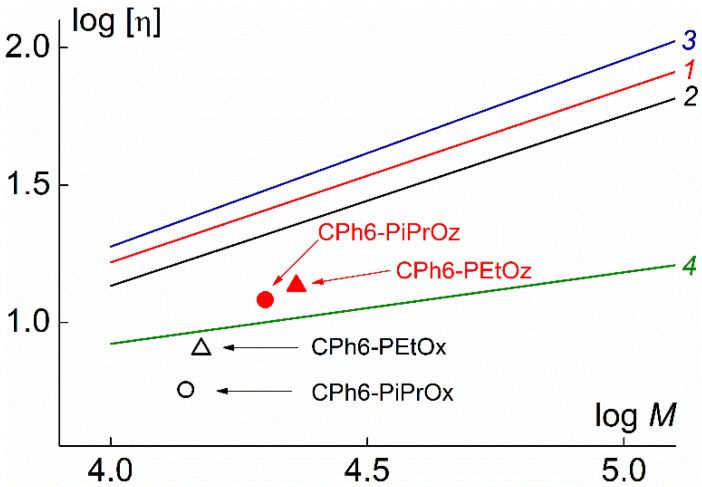
Intrinsic viscosity [η] vs. molar mass *M* for the studied polymer stars in chloroform and MKHS dependences for linear PEtOx (*1* [26] and *2* [59]), PMeOx (*3* [26]), and star-shaped eight-arm C8A-PS (*4* [53]).

In Figure 7, the values of characteristic viscosity [η] for the studied polymer stars are plotted as a function of MM. Additionally shown are the Mark–Kun–Hauwink–Sakurada (MKHS) dependences for linear PEtOx [26,59] and poly-2-methyl-2-oxazoline (PMeOx) [26], studied in thermodynamically good solvents. It is clearly seen that the points corresponding to [η] for CPh6-PAlOx and CPh6-PAlOz lie significantly lower than the straight lines for PEtOx and PMeOx. On the other hand, they are grouped around the MKHS dependence for eight-arm polystyrene with calix[8]arene core (C8A-PS) [53]. Low values of [η] indicate high intramolecular density of CPh6-PAlOx and CPh6-PAlOz.

**Figure 8:**
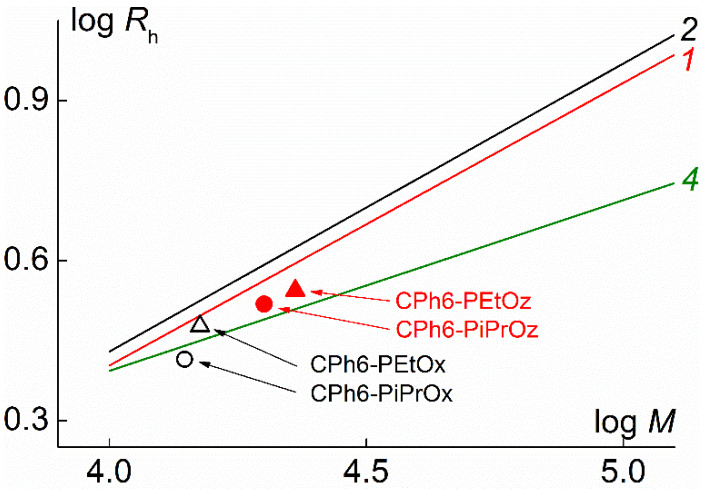
Hydrodynamic radius *R*_h_ vs. *M* for the studied polymer stars in chloroform and MKHS dependences for linear PEtOx (*1* [26] and *2* [59]) and star-shaped eight-arm C8A-PS (*4* [53]).

A similar conclusion can be made by comparing the molar mass dependences of the hydrodynamic radii *R*_h_ (Figure 8). For the studied CPh6-PAlOx and CPh6-PAlOz, the values of *R*_h-D_ are slightly less than *R*_h_ for linear PEtOx and are close to the hydrodynamic radius of C8A-PS molecules. The difference in *R*_h_ for the compared polymers is noticeably smaller than in the case of intrinsic viscosity. This is due to the lower sensitivity of the *R*_h_ value to the change in the shape of the molecules and the molar mass of the polymer compared to the [η] value [47].

To analyze the architecture dependence obtained from the experimental data, the so-called contraction factors, i.e., the rations of conformational and hydrodynamic parameters of the branched polymer to that of the linear polymer having the same molar mass are usually employed. Contraction factors are (*i*) ratio of the squared gyration radii *R_g_*:

*g* = (*R_g_*)*_br_*^2^/(*R_g_*)*_lin_*^2^ (5)

(*ii*) ratio of the intrinsic viscosities:

*g*′ = [*η*]*_br_*/[*η*]*_lin_* (6)

(*iii*) ratio of the hydrodynamic radii:

*h* = (*R_h_*)*_br_*/(*R_h_*)*_lin_* (7)

The subscript characters “br” and “lin” in Equations (5)–(7) mean that the parameter refers to the branched and linear polymer, respectively.

Unfortunately, the geration radii *R*_g_ for CPh6-PAlOx and CPh6-PAlOz could not be experimentally measured due to the small size of the macromolecules. The *g*′ and *h* values are shown in Table 4. They were determined using the MKHS dependences for linear PEtOx [26,59]. The number on the left in each cell of Table 4 was calculated by comparing the characteristics of CPh6-PAlOx and CPh6-PAlOz with the data obtained by Filippov and Hoogenboom [59]. To determine the values in the cells on the right, we used the dependences [η] and *R*_h_ on MM, established by Schubert and Nischang [26]. As can be seen from Table 4, the contraction factors for polyoxazoline stars are smaller than *g*′ and *h* for CPh6-PAlOz.

Note that we analyze the contraction factors by comparing the hydrodynamic characteristics of all the studied stars with the data for PEtOx. Therefore, the “true” values of *g*′ and *h* are obtained only for CPh6-PEtOx. For the remaining polymers, *g*′ and *h* shown in Table 4 will slightly differ from the values which could be obtained using the values [η] and *R*_h_ for linear PiPrOx, PiPrOz, and PEtOz to determine them. It can be expected that the differences in [η] and *R*_h_ for the linear polypeptoids under consideration are small. Compared to PEtOx, the side chains of PiPrOx and PiPrOz contain one –CH_3_ group more, and the monomer units of PAlOz are longer by –CH_2_– group. Obviously, the increase in the size of the side chain should be accompanied by an increase in [η] and *R*_h_, and elongation of the monomer unit of the flexible main chain can cause a decrease in these parameters. However, systematic studies of various classes of comb-shaped polymers [47] showed that, at the described structural variations, the hydrodynamic and conformational characteristics of polymers practically do not change. For example, for polymers with short side chains containing less than six bonds, the considered changes fall within the experimental error [47]. Therefore, the values of *g*′ and *h* given in Table 4 for CPh6-PiPrOx, CPh6-PiPrOz, and CPh6-PEtOz can serve as a reliable quantitative characteristic of these polymers.

Probably, Zimm and Kilb made the first attempt to find a relationship between *g* and *g*′ for star polymers [60]. Using the Kirkwood–Riseman approximation for the hydrodynamic interaction, they concluded that *g*′ = *g*^1/2^. However, such behavior has not been observed experimentally [61], and Kurata with coauthors established empirically that *g*′ = *g*^0.6^ [62]. Later Weissmuller and Burchard [63] showed that dependence of *g*′ on *g* cannot be described by a power law, and after analyzing the experimental data for a large number of polymer stars with various structures, they proposed the equation:

*g*′ = (1.104 − 0.104*g*^7^)*g*^0.906^ (8)

Stockmayer and Fixman [64] assumed that for diffusion *h* and viscosity *g*′ contraction factors are related by the formula:

*g*′ = *h*^3^ (9)

Using the above equations, we calculated the values of the contraction factor for the studied polymer stars from the data of rotational (*g*_η_, Equation (8)) and translational (*g*_h_, a combination of Equations (8) and (9)) friction (Table 4). Similar to *g*′ and *h*, the compression factors *g*_η_ and *g*_h_ are lower for CPh6-PAlOx than for CPh6-PAlOz. The values of *g*_η_ and *g*_h_ obtained for the studied polymers can be compared with the theoretically predicted *g*. For star-shaped polymers with very long monodisperse arms, Zimm and Stockmayer obtained [65]:

*g* = (3*f_a_* − 2)/*f_a_*^2^, (10)

where *f*_a_ is the arm number. Accordingly, for six-pointed stars, *g* = 0.44. For stars with polydisperse arms [66,67], the relation is

*g* = 3*f_a_*/(*f_a_* + 1)^2^, (11)

that is, for a star with six arms, *g* = 0.37. From Daoud–Cotton theory, we can get [68]

*g* = *f_a_*^−4/5^, (12)

therefore, for six-arm stars, *g* = 0.24.

The contraction factor *g*_η_, determined from the values of intrinsic viscosity, for a star with the shortest arm, corresponds to the conclusions based on the Daoud–Cotton theory. For other polymers, *g*_η_ are close to the *g* values for star-shaped macromolecules with long arms. The Kuhn segment length *A*_PEtOx_ for linear PEtOz is 1.4–1.8 nm [59,69]. Therefore, taking into account the above comments on the hydrodynamic and conformational characteristics of polypeptoids, it can be assumed that the studied polymer stars can be considered as long-arm stars, if the arms contain six or more Kuhn segments. As for the contraction factor obtained using translational friction data, for all polymers except CPh6-PiPrOx *g*_h_ exceeds theoretically predicted the *g* values. This discrepancy can be explained by the fact that when calculating *g*_h_ twice (both in Equation (8) and Equation (9)), the Gaussian chain approximation is used. In addition, a large experimental error should be taken into account when determining the hydrodynamic radii of macromolecules.

An important characteristic of the macromolecule behavior in solutions is the so-called hydrodynamic invariant [47,70,71]:

(13) $$A_{0}\equiv \eta_{0}{\left(\frac{D_{0}}{T}\right)}^{2/3}{\left[\frac{\left[\eta \right]s_{0}R}{100\left(1-\bar{v}\rho_{0}\right)}\right]}^{1/3}$$

For linear macromolecules, *A*_0_ is constant over a wide MM region: the average experimental values are *A*_0_ = 3.2⋅× 10^−10^ erg⋅K^−1^mol^−1/3^ for flexible chain and 3.8 ×⋅10^−10^ erg⋅K^−1^mol^−1/3^ for rigid chain polymers [47,70]. These are in good agreement with the theoretical values *A*_0_ obtained using various methods for considering the hydrodynamic interaction and using different statistical models of linear macromolecules [47,70]. Hyperbranched polymers and dendrimers are often characterized by very low, up to *A*_0_ = 2 ×⋅10^−10^ erg⋅K^−1^mol^−1/3^, values of the hydrodynamic invariant [50,72]. For linear PEtOz, the typical value for flexible chain polymers is *A*_0_ = 3.45⋅× 10^−10^ erg⋅K^−1^ mol^−1/3^ [26].

As can be seen from Table 3, *A*_0_ for CPh6-PAlOz are in good agreement with the experimental values of the hydrodynamic invariant for flexible chain polymers. For stars, CPh6-PAlOx *A*_0_ is noticeably lower; it is close to the theoretical value for impermeable hard spheres 2.88⋅× 10^−10^ erg⋅K^−1^ mol^−1/3^. It can be assumed that this difference is due to different arm lengths in CPh6-PAlOx and CPh6-PAlOz molecules.

### 3.3. LCST Behavior of CPh6-PAlOx And CPh6-PAlOz

**Figure 9:**
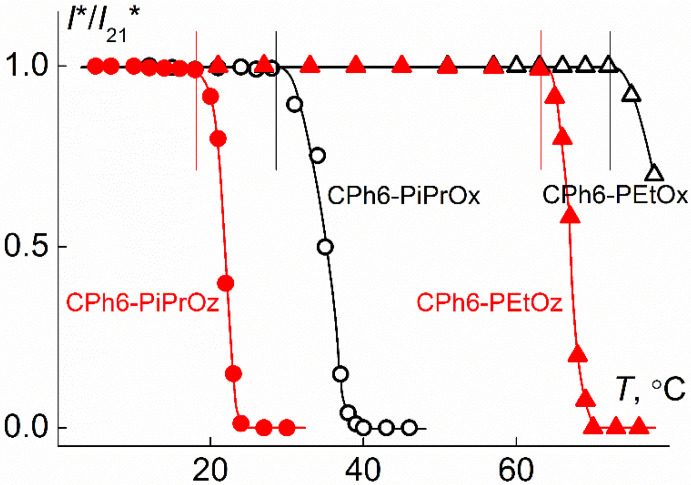
Dependences of relative transmittance I*/*I*_21_* for aqueous solutions of studied polymer stars. *I*_21_* is intensity of optical transmittance at *T* = 21 °C.

All synthesized stars exhibit LCST behavior, as illustrated in Figure 9. The cloud point temperature *T*_cp_ is defined as the beginning of the decline in optical transmittance. In both ranks *T*_cp_ for stars with isopropyl groups in the side chains of arms, lower than for polymers with ethyl groups. This behavior is in qualitative agreement with what is observed for linear PAlOx and PAlOz [35,73,74,75]. As in the case of linear polypeptoids, elongation of the monomer unit leads to a decrease in *T*_cp_: the difference in *T*_cp_ is about 11 °C for the pair CPh6-PiPrOx and CPh6-PiPrOz and 9 °C for CPh6-PEtOx and CPh6-PEtOz. However, in this comparison, it should be taken into account that MM CPh6-PAlOz is noticeably higher than MM CPh6-PAlOx, which can also lead to a decrease in the cloud point. As for the absolute values of *T*_cp_, for CPh6-PAlOx they are in agreement with the data for the star-shaped four- and eight-arm PAlOx with calix[n]arenes and carbosilane dendrimers as the core [20,28,46,76].

## 4. Conclusions

For the first time, star-shaped six-arm polypeptoids, namely, poly-2-alkyl-2-oxazine and poly-2-alkyl-2-oxazoline were synthesized successfully using cationic ring-opening polymerization. The derivative of hexaaza[2_6_]orthoparacyclophane with long enough undecenyl spacers was used as macroinitiators in core-first synthesis. NMR spectroscopy and chromatography confirmed the structure prepared samples and showed practically simultaneous polymerization of the arms on the initiation centers.

Using the methods of molecular hydrodynamics and optics, it was established that the arm structure does not affect practically the conformation of star-shaped macromolecules of studied polypeptoids. Much more important is the length of the arms, elongation of which leads to an increase in contraction factors. This behavior is explained by the close equilibrium rigidity of the polymers used as arms. The polymer stars under investigation are the long-arm stars, if the arms contain six or more Kuhn segments. On the one hand, the PAlOz and PalOx arms are folded, and star-chaped CPh6-PAlOz and CPh6-PAlOx are characterized by elevated intramolecular density in comparizing with their linear analogs. Changes in the conformational and hydrodynamic behavior of the studied polymer stars with decreasing of arm lengths are indicated by a decrease in the values of the hydrodynamic invariant.

The synthesized stars were thermosensitive exhibiting LCST behavior. At the passage from CPh6-PAlOx to CPh6-PAlOz, a decrease in the temperature of phase separation is observed, which is in accordance with the behavior of linear analogs. The cloud point temperature for CPh6-PEtOz is higher than the *T*_cp_ value for its structural isomer CPh6-PiPrOx.

## Figures and Tables

**Table 1:** Hydrodynamic characteristics and molar masses of investigated polymer stars.

Polymer	*s*_0_, Sv	*R*_h-D_, nm	$\bar{v}$ cm^3^g^−1^	*M*_sD_, g⋅mol^−1^	[η], cm^3^g^−1^	*dn*/*dc*, cm^3^g^−1^
CPh6-PEtOz	2.7	3.5	0.843	23,000	13.6	0.088
CPh6-PiPrOz	3.6	3.3	0.890	20,000	12.1	0.073
CPh6-PEtOx	2.3	3.0	0.861	15,000	8.0	0.091
CPh6-PiPrOx	2.3	2.6	0.851	14,000	5.7	0.084

**Table 2:** Molar masses and structure characteristics of synthesized polymer stars.

Polymer	*M*_sD_, g⋅mol^−1^	*ω*, mol %	*M*_0_, g⋅mol^−1^	*N*_tsc_	*L*_tsc_, nm	*L*_arm_, nm
CPh6-PEtOz	23,000	7.2	113	31	15.9	17.1
CPh6-PiPrOz	20,000	8.3	127	24	12.1	13.4
CPh6-PEtOx	15,000	11.0	99	22	8.5	9.8
CPh6-PiPrOx	14,000	11.8	113	18	6.9	8.1

**Table 3:** Hydrodynamic characteristics of investigated polymer stars.

Polymer	*L_arm_/R_h-D_*	*L_arm_/A_PEtOx_*	*A*_0_⋅10^10^, erg⋅K^−1^mol^−1/3^
CPh6-PEtOz	4.9	11	3.3
CPh6-PiPrOz	3.8	8	3.2
CPh6-PEtOx	3.3	6	2.8
CPh6-PiPrOx	3.2	5	2.8

**Table 4:** Contraction factors for investigated polymer stars.

Polymer	*g′*	*h*	*g_η_*	*g_h_*	*L_arm_/A_PEtOx_*
CPh6-PEtOz	0.60/0.49	0.88/0.94	0.51/0.41	0.58/0.74	11
CPh6-PiPrOz	0.58/0.47	0.87/0.93	0.49/0.39	0.57/0.71	8
CPh6-PEtOx	0.38/0.46	0.81/0.86	0.31/0.38	0.44/0.54	6
CPh6-PiPrOx	0.28/0.34	0.71/0.76	0.22/0.27	0.29/0.36	5

## Supplementary Materials

The following are available online at https://www.mdpi.com/2073-4360/12/4/800/s1, Figure S1. 1H NMR spectrum of trianglamine; Figure S2. 1H NMR spectra of star-shaped poly(2-ethyl-2-oxazoline) (1), poly(2-isopropyl-2-oxazoline) (2), poly(2-ethyl-2-oxazine) (3), and poly(2-isopropyl-2-oxazine) (4). Solvent – d-chlorophorm.
