# Supplementary material for: Synthesis and Conformational Characteristics of Thermosensitive Star-Shaped Six-Arm Polypeptoids
Source: Polymers (Basel). 2020 Apr 3;12(4):800. doi: 10.3390/polym12040800 (PMC7240544; doi:10.3390/polym12040800)
Supplement: Supplementary file 1 [file polymers-12-00800-s001.pdf]

Supplementary Materials

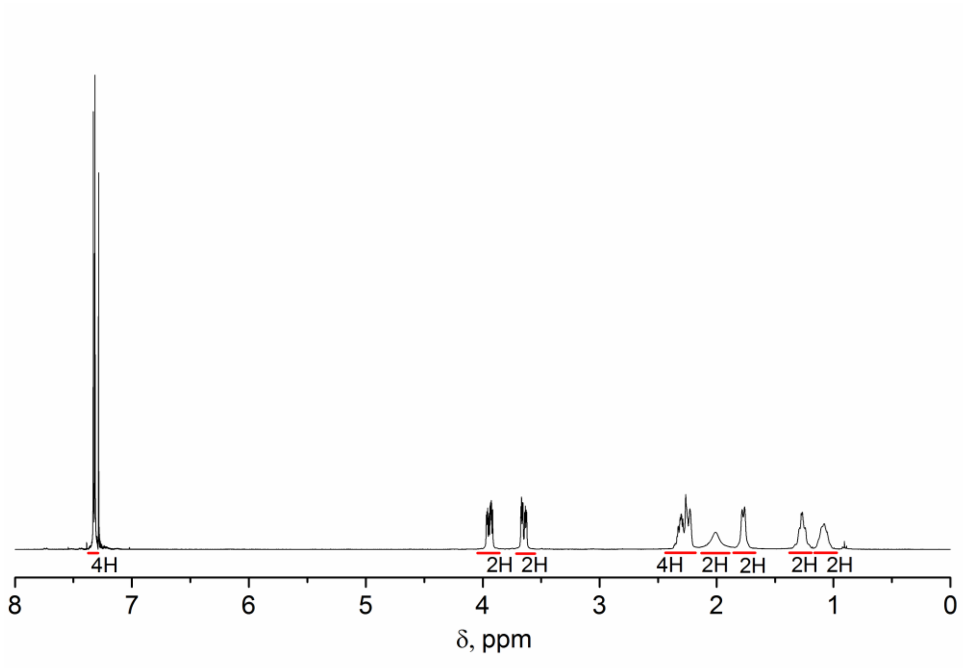

Figure S1. ^{1}H NMR spectrum of trianglamine.

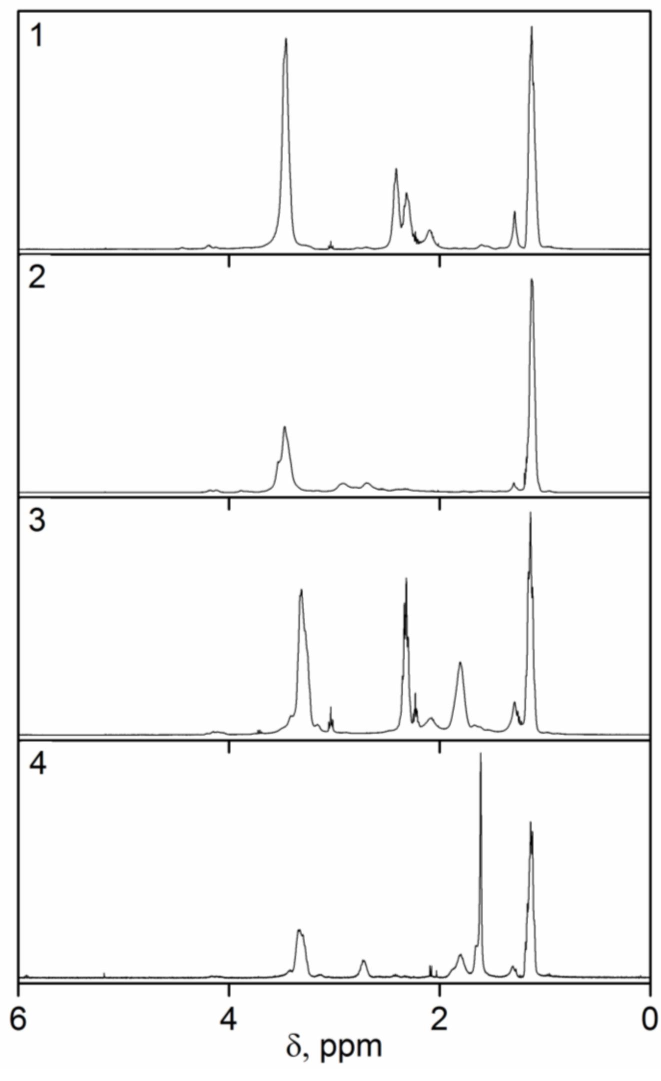

Figure S2. ^{1}H NMR spectra of star-shaped poly(2-ethyl-2-oxazoline) (1), poly(2-isopropyl-2-oxazoline) (2), poly(2-ethyl-2-oxazine) (3), and poly(2-isopropyl-2-oxazine) (4). Solvent – d-chlorophorm.
